# Multi-inflammatory syndrome in children (MIS-C) associated with COVID-19: a nursing perspective experience report from a high-income tertiary paediatric hospital context

**DOI:** 10.1186/s12969-023-00786-y

**Published:** 2023-01-18

**Authors:** Henrik Hjelmgren, Karin Andersson, Jessica Widegren, Erika Bergman, Anna Vermé, Karina Mördrup, Marcus Öhlander, Cecilia Bartholdson

**Affiliations:** 1 Department of Women’s and Children’s Health, Karolinska Institutet, Stockholm, Sweden; 2 Highly Specialised Paediatric Medicine and Orthopaedics, Astrid Lindgren’s Children’s Hospital, Karolinska University Hospital, Stockholm, Sweden

**Keywords:** Children, Clinical nursing records, Recommendations, MIS-C, Nursing status, Qualitative and quantitative methods

## Abstract

**Background:**

In the midst of the COVID-19 coronavirus pandemic, a new disease that affects children has arisen called *multisystem inflammatory syndrome in children* (MIS-C). Several research articles focusing on its medical aspects have been published, but very few have focused on nursing care. The aim of this study was therefore to describe the nursing status of children suffering from MIS-C and the experiences of registered nurses (RNs) in caring for these children in paediatric hospital inpatient care.

**Methods:**

The study design includes both quantitative nursing clinical record data and qualitative interview data. Quantitative data from the clinical records were analysed using descriptive statistics. Qualitative data analysis of the interviews was conducted using both deductive and inductive approaches with content analysis.

**Results:**

In total, 47 clinical records from children with MIS-C were investigated during January–March 2021. The mean age of the children was 8.8 years. Boys were more affected than girls. Challenges in children’s nursing status were related to circulation (fever and swelling), nutrition (great thirst and loss of appetite), pain, and psychosocial situations. When caring for children with MIS-C, nurses experienced “frustration over uncertainty of care”, “children’s illbeing” and “unavoidable procedures”.

**Conclusion:**

This study contributes knowledge to the ongoing nursing care of children suffering from MIS-C. The results show many different areas of nursing focus, which challenges nurses and other disciplines within paediatric hospital care. One important factor when caring for these children was the use of a central venous line early in the care process, which improved the quality of care. Moreover, the care of children suffering from MIS-C demands resources and time from healthcare professionals, especially RNs, to meet caring needs and reduce illbeing.

**Supplementary Information:**

The online version contains supplementary material available at 10.1186/s12969-023-00786-y.

## Background

### COVID-19 in children and MIS-C

Most children diagnosed with COVID-19 have mild or no symptoms [1]. However, in April 2020, the first reports on a new paediatric multisystem inflammatory syndrome (MIS-C) appeared [2]. Beginning with paediatricians Twittering on social media and then being described in newspapers and scientific reports, the new disease has challenged paediatric healthcare. There are three definitions of MIS-C: those of the Centers for Disease Control and Prevention, the World Health Organisation, and the Royal College of Health Paediatrics and Child [3]. There is no single test or symptom for diagnosing MIS-C; instead, the definitions include a group of symptoms and laboratory results as well as radiology findings [3]. Most children diagnosed with MIS-C report no background disease and were previously healthy [4]. Comorbidities are rare, apart from obesity (25.3%) [3]. Among children, no specific risk group has been found and MIS-C occurs in all ages from 0–19 years [5]; nevertheless, a recent review reveals a median age of 8 years and epidemiological enrichment for males (58.9%) and ethnic minorities (37.0% Black) [3]. Medical studies show that MIS-C has many symptoms similar to Kawasaki’s disease but has several differing characteristics, such as more intense inflammation [6]. The symptoms of MIS-C usually appear 2–6 weeks after infection with COVID-19 and the severity of the illness varies [7]. Children suffering from MIS-C present with fever (99.4%), gastrointestinal (85.6%) and cardiocirculatory issues (79.3%), as well as increased inflammatory biomarkers. However, 50.3% present with respiratory symptoms as well. More than half of patients suffer from shock and multiorgan impact; cytokine storm is present in critically ill children and demands treatment and preparedness for intensive care [3]. Many children (73.3%) need intensive care treatment, but mortality is rather low (1.9%) [3] compared to adults and elderly people with COVID 19 respiratory illness. In Sweden, around 280 children diagnosed with MIS-C have been reported as of November 2021. While the degree of illness has varied, fortunately, no children have died; however, around 25% have needed intensive care. A newly published report showed that the absolute risk for MIS-C were very low, as the incidence rate was 6.8 per 100 000 person-years [8]. At hospitals, all symptoms are documented in the child’s clinical record by the involved health care professionals (HCPs).

### Clinical nursing records

In Sweden, the definition of clinical nursing records is data recorded by registered nurses (RN) in the clinical record concerning patients’ nursing status, including various symptoms and nursing care given to the patient and judgment of the patient’s progress. It has been mandatory for RNs in Sweden to record health status since 1986 [9], and since 1991, the validated VIPS model framework has been used and tested in many areas of nursing care, including paediatrics [10, 11]. VIPS is an acronym for the Swedish terms Well-being, Integrity, Prevention and Safety. This framework provides support, helps to structure the information in the clinical nursing record and allows HCPs easy access for clinical audits [12]. Clinical audits are a way for HCPs to seek to improve care and evaluate their own practices [13]. The VIPS model is now used for recording nursing status and care not only in Sweden, but also in Denmark, Norway, Estonia and Latvia [10]. So far, very few studies have reported evidence concerning nursing status and care related to children affected by MIS-C. Observations of nursing status and documentation add valuable insight to the holistic picture of children suffering from MIS-C.

## Aims

The aims of this study were to describe the nursing status of children affected by hyperinflammation MIS-C disease and RNs’ experiences of caring for these children in paediatric inpatient hospital care.

## Methods

### Design

In this study both quantitative clinical nursing record data and qualitative interview data were used.

### Setting of the study

All patients in this study were cared for at a Swedish university hospital with an integrated children’s hospital. The children’s hospital comprises various medical and nursing areas, such as neurology/orthopaedics/rheumatology. Specifically, the children’s hospital is organised into 20 patient flows and approximately 160 patient groups. This means that there is access to different care facilities, including paediatric intensive care and specialised inpatient and outpatient care.

### Data collection

Data collection was carried out using two different methodologies. First, three of the authors KA, JW and EB conducted audits of all the clinical nursing records of children affected by MIS-C admitted to the hospital during the period of Jan–March 2021. In the audits, children’s characteristics and VIPS keywords [11, 12] essential in paediatric care were used; data were transferred to a Microsoft Excel sheet. Second, focus group interviews were conducted by HH, KA, MÖ and CB with nurses who had gained experience in caring for children with MIS-C in two different paediatric wards at the children’s hospital. A semistructured interview guide was conducted by the first author (HH), and then discussed and revised by the co-authors, CB, KA and MÖ (Additional file 1). Each interview started with open-ended questions, followed by probing questions to elicit more elaborative answers [14]. The VIPS model was then used deductively, and the participants were asked questions concerning the fixed keywords in the VIPS model. This was done to ensure that all nursing aspects were covered.

### Sampling

Children eligible for the study were identified via the Swedish Paediatric Rheumatology Register [15]. The eligibility criteria were children aged 0–17 years who were diagnosed with MIS-C and cared for at an inpatient care unit at the children’s hospital. For the qualitative data, purposive sampling was used to gather participants who could generate rich, informative data and in-depth information about the experiences of caring for children with MIS-C [14].

### Data analysis

Quantitative data were analysed with descriptive statistics by calculating the numbers and percentages from the audits in the clinical nursing records. The qualitative analysis of the interviews was conducted using both deductive and inductive approaches with content analysis, as described by Elo and Kyngäs [16, 17]. The audio-recorded interviews were transcribed into digital documents. The VIPS model was used as a framework for deductive analysis. The inductive analysis was performed in several steps: extracting units of meaning, coding, writing memos and abstraction of the content. The emerging categories and subcategories of RNs’ experiences of caring for children with MIS-C were summarised and described in relation to the transcribed data. The first and last authors analysed the data separately and discussed the categories before writing the results section. All the authors were involved in the analysis by discussing the categories and their content in relation to the whole.

## Results

### Quantitative data from clinical nursing records

In total, 47 clinical nursing records from children with MIS-C were investigated, focusing on characteristics of children diagnosed with MIS-C and the VIPS keywords corresponding to essential paediatric nursing status, i.e. breathing, circulation, elimination, nutrition, pain, psychosocial and sleep. The mean age of the children was 8.8 years. Boys 33 (70.2%) were more frequently represented than girls 14 (29.8%), and most of the children were previously healthy. One-fifth of the children had some kind of respiratory condition, for example, infection-induced asthma. Four children (8.5%) needed care in the intensive care unit (Table 1).

**Table 1:** Characteristics of children diagnosed with MIS-C at the children’s hospital from Jan–March 2021

Characteristics	All patients *N* = 47 (%)
***Age***	
*Mean*	8.8 yr
*Median*	8 yr
< *1 yr*	2 (4.3)
*1–4 yr*	8 (17.0)
*5–9 yr*	17 (36.2)
*10–15 yr*	20 (42.6)
***Gender***	
*Boys*	33 (70.2)
*Girls*	14 (29.8)
***Underlying conditions***	
*Previously healthy*	36 (76.6)
*Respiratory*	7 (14.9)
*Obesity*	1 (2.1)
*Other*	3 (6.4)
***Hospitalisation***	
Inpatient care	
≤ *3 days*	5 (10.6)
*4 days*	6 (12.8)
*5–9 days*	24 (51.0)
≥ *10 days*	12 (25.5)
*Need of intensive care unit*	4 (8.5)

Some symptoms were present in most children. These were: fever more than 5 days presented in 34 (72.3%) of the children; lack of appetite and pain symptoms in 37 (78.7%) and 35 (74.5%) of the cases, respectively (Table 2).

**Table 2:** Description of the nursing status of the included children

Number of clinical records (*N* = 47)	
**VIPS Keyword**	**n (%)**
***Breathing***	
In need of oxygen	11 (23.4)
***Circulation***	
Fever ^*^duration	
≤ *3 days*	8 (17.0)
*4 days*	5 (10.6)
≥ *5 days*	34 (72.3)
***Elimination***	
*Vomit*	19 (40.4)
*Diarrhoea*	25 (53.2)
***Nutrition***	
Lack of appetite	37 (78.7)
***Weight***	
≥ *5*% weight gain	11 (23.4)
≥ *3*% weight loss	4 (8.6)
None measured weight during hospital stay	3 (6.4)
Pain^**^	35 (74.5)
***Psychosocial***	
Fear of needles	8 (17.0)
Sad and scared during nursing procedures	22 (46.8)
***Sleep***	
Disturbed sleep^***^	10 (21.3)

^*^Fever was defined as a body temperature > 38.5

^**^Number of children who had pain documented in the clinical nursing record

^***^Based on the child’s normal sleep patterns

### Qualitative data from focus group interviews

In total, 11 RNs participated in one pilot interview and three focus group interviews. Data from the pilot interview are included in the results since the data were well-suited to the purpose of the study. Analysis of the demographic data of nurse participants showed that the age range varied from 22 to 47 years and length of work experience varied from 9 to 168 months, i.e. 14 years (Table 3).

**Table 3:** Demographics of participants (nurses) in the interviews

	Participants (n)	Mean age (Min–max)	Paediatric ward	Academic degree BSc/MSc	Length of work experience Mean (min–max)
Pilot interview	1	27	Infectious/medicine	BSc	32 months
Focus Group 1	4	29 (22–45)	Infectious/medicine	BSc, MSc	51 months (10–168)
Focus Group 2	3	35 (25–47)	Neurology/orthopaedic/rheumatology	BSc, MSc	76 months (24–156)
Focus Group 3	3	28 (27–30)	Neurology/orthopaedic/rheumatology	BSc, MSc	37 months (9–78)

### Perceptions of nursing status using the VIPS model framework

In this section, additional descriptive nursing statuses and those included from above are presented as they are complementary to the quantitative audits of the clinical records and not feasible to describe in numbers.

#### Activity

Many of the older children were described as not being able to stand up when experiencing the worst hyperinflammation process. The RNs stated that the children expressed pain when trying to walk and that it was almost impossible to get the children out of bed. The patients suffered from fatigue and were often extremely tired but still needed to get up to control their weight and visit the toilet.

#### Breathing

The participants described breathing as not a major concern for the children. Some children suffered from minor coughing and reduced oxygen saturation. Often, their respiration rate was high due to the high fever.

#### Circulation

The RNs were concerned about the children’s circulation status, which often drew their attention. High fever and swelling were major concerns that needed the nurses’ consideration and/or treatment with medicine. The RNs thought that the children perspired a lot due to fever, and that could affect the circulation as well.

#### Communication

The participants described some of their patients as having cognitive symptoms, such as hallucinations and other neurological changes. The children often became unusually angry, so that their parents did not recognise their child.

#### Coordination

The RNs needed to plan daily care, as well as discharge and home care, even when they were uncertain about the child’s progress. Planning was carried out with various paediatricians and rheumatology specialists. Due to physicians’ workload, the medical rounds were often very late, leading to challenges in the coordination of the care given.

#### Drug administration

The RNs described the administration of drugs for children with MIS-C as stressful. There were also painful injections and infusions for the children. The RNs spent a lot of time preparing and administering drugs.

#### Elimination

The RNs described elimination control challenges for children without urine catheters. None of the RNs mentioned any problems with the children’s faeces. In the early admission stage, the RNs found that children often vomited, but that it often stopped upon arrival at the ward.

#### Nutrition

The children were described as having a loss of appetite and being so tired that they did not have the energy to eat or drink. Some of the children were extremely thirsty, which was not easy to cope with, as there were often regulations on intake.

#### Pain

The uncertainty of the location of pain and how to handle it were often discussed in the interviews. It was difficult for the RNs to interpret the pain. The RNs were uncertain as to whether the children cried out of fear of HCPs and the procedures or if it was due to distinct pain. When defined, the RNs often observed pain from the skin, head, stomach or muscles and joints. The children were listless and uncomfortable.

#### Psychosocial

The children were often described as having anxiety and fear of upcoming procedures, especially needle-related ones. The RNs perceived that this was related to the frequency of needle-related procedures. Worried parents were also a major concern for the RNs. They described families in crisis and parents trying to cope with a previously healthy child who was now severely ill. The parents had many questions to which the HCPs did not have an answer. In most cases, the RNs contacted a social worker to facilitate the parents’ difficult situation.

#### Skin

The RNs related that the children had pain from the skin, as it was burning. The skin was also swollen and could have red rashes, which some children scratched so much that they caused wounds. This often led to difficulties in the success of insertion of periphery intravenous cannulas and blood sample collection, leading to worsening the situation and increasing illbeing.

#### Sleep

Disturbed sleep for the child was regularly mentioned. This was due to the many controls of vital parameters and medicines that had to be given during both day and night.

### RNs’ experiences of caring for children with MIS-C

**Fig. 1:**
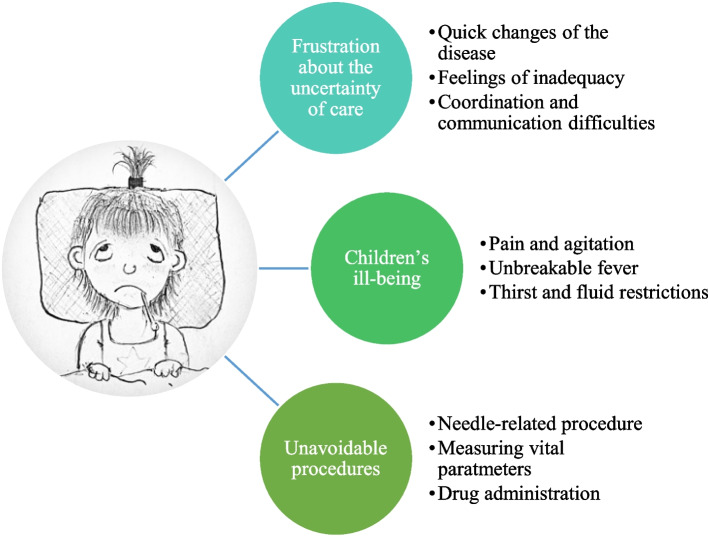
Overview of the categories and subcategories from the inductive analysis. (Picture drawn by Ingrid Hjelmgren.)

Three main categories and nine sub-categories were generated from the interviews (Fig. 1).

### Frustration over uncertainty of care

The RNs were frustrated by insufficient knowledge about MIS-C and that no one knew what to expect. Frustration was also related to the quick changes of the disease, feelings of inadequacy and coordination and communication in care.

### Quick changes in the disease

The RNs pointed out that the children could quickly get worse, which made the nurses stressed and worried. The main frustration was about not being able to predict when a child would worsen, resulting in the need to be alert at all times. The children demanded a lot of time for nursing care, and the nurses needed to be really focused.

*“And I think a bit about how fast it can go, that they become very ill, from the fact that they are admitted with fever and nausea and kind of rash to becoming very, very ill…” (Focus group 3)*

The nurses also related that these quick changes led to “heavy workload”, due to complex care needs. They reported that the children had procedures that had to be done all the time. The workload was described as similar in amount and type to that of intensive care. Even for experienced RNs, the severity of the illness and the quick changes were frustrating and emotionally distressing.

The care of the children could vary greatly depending on where they were in the course of the disease. The initial care phase differed a lot from the end phase, e.g. before discharge. After a couple of days, the children were feeling better due to the effects of the treatment, and the RNs could get some relief.

*“It’s usually a little different, someone is super ill and someone else is like [better], and if you discover [MIS-C] quite early and as soon as you give the Privigen (drug) it feels like it goes well quite quickly, but if it takes a while it feels [that the children] can get really bad and then I think it’s worse, then it’s a lot of things to do. If you are in there, you are stationed.”. (Focus group 1)*

#### Feelings of inadequacy

If the RNs cared both for children with MIS-C and for other children, they had to plan their working shifts very carefully to keep up with all the assignments they had during the day. However, even if well planned, feelings of inadequacy arose.

*“If it is in the beginning, then you can not have two MIS-C children yourself or, like, you can have one child with MIS-C but not two or three, because then you do not have time to do what you should…”(Focus group 3)*

Another aspect of inadequacy that the RNs experienced was the difficulty in giving information. It was not easy to know what to say to the children and their parents when everyone was uncertain of what would happen. However, the parents seemed to understand this difficulty.

*“…and I think they [parents] understand… that still COVID is also new to everyone, so I think they understand that they will not get more information, because we know nothing more than what we say”. (Focus group 2)*

Furthermore, inadequacy was about not being able to relieve children´s suffering for example, unlocated pain.

*“Well, pain, I think. That they just scream like, oh well, just where, where do they have pain, I do not know. Anyway, it has been experienced as a huge inadequacy and I do not know how to do it better for the children and then just like give a lot of pain relief without knowing where the focus is. It also feels not good. I would like to know so I can do better, too.” (Focus group 1)*

The inadequacy was also about not knowing the prognosis and what would happen next. After discharge, the RNs carried this uncertainty with them.

#### Coordination and communication difficulties

The participants described difficulties with the coordination of medical rounds. The physicians working with paediatric rheumatology were the ones assigned children with MIS-C, but there were very few such physicians, resulting in a heavy workload and poor availability, which led to late rounds.

*“But the rheumatologists, it feels like they have been too few. So they have worked very hard and it has been noticed, their phone rings a lot and such. I almost feel sorry for the rheumatologists because they have been so laboured, and they really have…or many have really done their best.” (Focus group 3)*

Nurses also described frustration that communication between nurses and physicians sometimes failed. The failure was derived from not understanding one another. One example of this was the blood sampling procedures. Nurses found it very hard to take repeated blood samples because the children suffered so much and because of the difficulties in blood sampling related to swelling and poor skin. Physicians, on the other hand, needed to have more information from blood tests to understand the child’s health status and how to treat it. Nurses described feelings of frustration related to negotiating with physicians as to how often and how much blood they were to take.

*“Right at the beginning, I also think before we understood what happened and before the doctors understood, it was as if you got from the doctors to give a little sedative and just take the samples. Yeah, right, but it is not possible.” (Focus group 2)*

### Children’s illbeing

The RNs experienced the children with MIS-C as extremely ill. The illbeing comprised both physical and psychological elements. Children were perceived as having issues regarding pain, fever, swelling, thirst and fatigue, and they were unhappy and needed a lot of support.

#### Children’s pain and agitation

The RNs stated that the children had pain that was experienced as untreatable. The children were screaming, and the RNs felt feelings of insufficiency. Furthermore, the RNs related that the children had pain throughout their bodies with itchiness and feelings of unidentified discomfort.

*“And the pain in the whole body…I had a patient who described it as if the arm felt like it ran ants on it and tickled the whole body and that it just kind of sparkled. Yeah, like anxiety and pain in the body, as well. And I tried to ask what do you want to do, and in the end he just said, ‘No, you cannot do anything’.” (Pilot interview)*

The RNs closely observed the children and found that they were often agitated and afraid. This often led to the children having difficulties participating in their care and sometimes large problems related to cooperation.

When the RNs tried to do procedures such as periphery venous catheters, blood pressure and weight control, the children also felt pain and anger.

*“And then it was like this that once you managed to put a Peripher vein catheter [PVC], then the children were so angry and upset, so then they pulled it [PVC] off.” (Focus group 3)*

#### Unbreakable fever

For most of the children, the RNs observed an intense, unbreakable fever that could often not be reduced by antipyretics. The skin was untouchable due to pain and swelling, which made care difficult. Cold blankets sometimes reduced the burning fever.

*“…when a child who has been there as well as been very ill, you are not even allowed to touch their skin, well those children I think they are burning”. (Pilot interview)*

#### Children’s thirst and fluid restrictions

It was challenging for the RNs to control the children’s fluid balance because the fluid balance status in MIS-C patients could shift very quickly. It was also challenging to control intake, as the children became very thirsty but needed restrictions. One RN described the situation as follows:

*“Well, also this with those children who had such a failure in their fluid balance. I had a little boy who, like, every time we met him, he screamed for a glass of water, so you stand next to him and just, like, this I cannot give because you [the boy] have already drunk too much. And you [the boy] are so dehydrated and well you [the nurse] feel like the devil himself…” (Focus group 1)*

### Unavoidable procedures

Something the RNs discussed concerning children with MIS-C was that they underwent many unavoidable procedures that contributed to major challenges in care. There was no choice; the procedures had to be done, no matter what.

#### Blood sampling and venous access

The large number of needle-related procedures in the care of these children was very difficult for the children and frustrating and challenging for the RNs. Venous access that broke and collapsed veins made it difficult for the RNs, and it was not easy for the RNs to make it bearable for the children. If the child lacked venous access, it often worsened the situation for many reasons.

*“But I probably still think that the hardest thing, the absolute hardest thing, about these children is the blood sampling procedures, because if we do not have access, it’s awful. So, if we do not have venous access that allows aspiration of blood or you have to puncture them every day. Oh my God, that was probably the biggest problem with all these patients, I think.” (Pilot interview)*

The blood sampling procedures were often demanding due to the illness itself and the many blood analyses required, which needed special attention and led to several repeated punctures for the child.

When a new guideline was created, the RNs experienced great relief. In the guideline it was stated to prioritize a central venous line which eased blood sampling and drug administration procedures.

#### Measuring blood pressure and weight

Measuring blood pressure was experienced as very demanding for the children. RNs described this as a terrible situation because the children suffered a great deal of pain from it.

*“The children perceive measuring blood pressure as awful. Almost everyone thinks that it is awful, that it tightens around the arm, and hurts so incredibly.” (Pilot interview)*

RNs also related that it was hard and stressful to push the children to stand on a scale or sit on a scale chair, for example, when the child was very weak and ill. Due to the importance of detecting fluid retention, RNs found that they had no choice and that the procedures were unavoidable.

#### Drug administration

The RNs had a lot to think about regarding the complex drugs in care and they feared the risk of making mistakes. For example, MIS-C treatment included unfamiliar special drugs, which were challenging and created frustration. Challenges related to drug administration also included the amount of time required to prepare and provide the drugs.

*“Infusions that take an hour, infusions that take three hours, it beeps all the time, they get the anticoagulant subcutaneously twice a day, they get, like, difficult medications or what to say …” (Focus group 2)*

## Discussion

This study aimed to contribute knowledge to the ongoing care of children suffering from MIS-C. The main findings from audits of clinical records were that boys were more frequently diagnosed with MIS-C, and the RNs observed that children had difficulties related to circulation (fever and swelling), nutrition (great thirst and loss of appetite), pain, and psychosocial situations. Furthermore, the main findings from the interviews included RNs’ experiences of frustration about the uncertainty of care and children’s illbeing as well as unavoidable procedures.

RNs caring for children suffering from MIS-C had to cope with the uncertainty of care, which created frustration and stress. When a new disease arises, a major workload is placed on RNs. In a study concerning Kawasaki disease, similar to MIS-C, RNs played an important role in controlling symptoms and vital parameters [18]. It is likely that the RNs’ perceived responsibility for the children’s symptoms and vital parameters was a major factor correlating to stress. In a previous report from RNs working in South Korea during the Middle East Respiratory Syndrome outbreak, RNs experienced unclear and frequently changing guidelines as stress-inducing [19]. Several RNs in the present study described the situation as stressful on multiple levels: stress over the new disease with a severely ill (previously healthy) child, stress over the new workload and stress over not having a concrete caring plan. Nurses have previously reported stress related to lack of evidence-based treatment [20]. MIS-C treatment and care at our hospital where improved when paediatricians with a specialty in rheumatology assumed primary responsibility. A multidisciplinary guideline were made and it was decided that these patients were prioritised with central venous line access early in their treatment process.

As in previous studies, the children in this study were primarily boys, and the average age was around 9 years [21, 22]. The hospitalisation times for children with MIS-C were longer, 5–9 days (51%), compared to the other patients at the Children’s Hospital, which was approximately 3 days. In our cohort, 8.5% needed intensive care. In a previous systematic review, significantly higher numbers of children were reported (73%) [3]. This could be explained by the very advanced care that was provided at the inpatient units at our Children’s Hospital, also corresponding to nurses’ descriptions in the interviews that the care provided was equivalent to that of intensive care as they mentioned they were “stationed” in the patient room.

Two of the major difficulties identified in nursing status from the clinical records and interviews in our study were children’s circulation and pain. Most often, pain was described as originating from the whole body, resulting in difficulties in identifying and locating it. In a recent study, neck pain and retropharyngeal oedema were described as associated with MIS-C [23]; nevertheless, in our study, neck pain was not specifically observed by the RNs. In other studies, abdominal pain and headache were reported [7, 24, 25], which correlates with what the RNs in our study described.

Not only did pain occur due to the illness itself, but it also occurred in relation to unavoidable nursing procedures, such as venous catheter insertion. One major task for nurses is to balance and prioritise these procedures. In a study about ethical issues in paediatric care, painful procedures were found to be ethically difficult [26]. It is reasonable to assume that nurses in our study found it ethically difficult when they knew that they had to expose children to pain by performing unavoidable procedures, especially when the children did not cooperate. To perform painful/unpleasant procedures on children who resist such treatment has also been found to be morally distressing in paediatric care [27]. Further research could focus on the ethical aspects of caring for children suffering from a rather unknown disease such as MIS-C. Future research could also include the perspectives of paediatricians who care for these children. The RNs in our study described paediatricians heavy workload and stress, which resulted in late rounds and delayed treatment decisions, which would likely expose them to ethical questions and moral distress.

In our data RNs described that the children were angry or agitated, which also relates to the neurological manifest symptoms that MIS-C has, which complicates nursing care further. Moreover, the analysis in our study revealed that nearly half of the patients were sad and experienced fear in connection with nursing procedures. This finding is consistent with a recent systematic review and meta-analysis related to the fear of needles [28]. It was found that prevalence estimates for needle fear ranged from 20–50% in adolescents [28]. Sometimes, it is not easy to determine whether a child is crying out of pain, fear or both. When caring for children suffering from an unknown disease like MIS-C, it will most likely be important to take numerous blood tests frequently. In these cases, we would argue for the early insertion of a central venous catheter to reduce both pain and fear, provided that it is medically safe.

Three-quarters of the children in our cohort suffered from fever for more than 5 days, which is in line with previous research [3]. Fever interestingly often has an impact on other symptoms as well. According to our experience, in this study high fever could accompany pain from the skin. Research has found that children with severe symptoms of MIS-C often suffer from both fever and pain [24]. Therefore, it is crucially important for HCPs to reduce fever to decrease different kinds of pain. Moreover, it is important to reduce fever to increase children’s appetites. In our cohort, almost 80% of the children suffered from a loss of appetite, which also probably can be closely linked to the gastrointestinal symptoms suffered by many of the children [29].

A further challenge that the nurses described was the combination of children’s thirst and fluid restrictions. This was combined with a situation in which parents were present in the children’s rooms at all times and were therefore the ones registering fluid intake. Most parents managed it perfectly, but in some cases, it was challenging for the nurses to know whether the fluid intake was adequately registered, which created uncertainty among the nurses. Previous research has shown that parents play a very important role in paediatric care and that forming a partnership of trust is key [30]. It is essential that parents have the possibility to be “only” parents while assisting in general care actions, such as fluid intake registration. Research has shown that parents want to take part in their children’s care but according to their own preferences [31]. We argue that it is important to have a family-centred approach and thereby strive to understand what is best for each individual child and parent from their perspective, protect the parents’ autonomy and promote parents as active partners while confirming their experiences. However, the challenge remains if there is a heavy workload on nurses and parents must assist in general care activities even when they do not want or have sufficient resources to cope with it.

To summarize, it is clear that HCPs face multiple challenges when caring for children affected by MIS-C. In order to deal with these challenges and contribute to knowledge to all HCPs we have put together a suggested list of concluding recommendations for care. These concluding recommendations derive from data and are based on nursing status and observations found in this study (Table 4).

**Table 4:** Nursing status, observations and recommendations of care related to children suffering from MIS-C

Nursing status	Observations	Recommendations of care
- Activity	Fatigue	Coordinate care to facilitate rest
- Breathing	Decreased saturation High respiration rate	Oxygen when needed, antipyretics when high respiration rate in connection to fever
- Circulation	High fever, swelling	Add cold blankets to antipyretics Strict monitoring of fluid intake and elimination
- Coordination/ - communication	Heavy workload Poor communication Agitated children	Decrease patient–nurse ratio during the first critical phase The interprofessional team is very important, looping in at the end of the day to evaluate team work
- Drug administration	Time consuming preparation and administration	If not possible to decrease patient–nurse ratio, add a pharmacist to the team
- Elimination	Control challenges	Controlling elimination is very important; if the children and parents cannot document quantities, there must be a clear plan as to who is responsible for this
- Nutrition	Loss of appetite Great thirst	Nutrition plan, together with a dietician. Optimise food and fluids within the confines of the restrictions
- Pain	Difficult to locate	Act to have a pain strategy plan that includes clearly addressing multiple pain locations as well as monitoring and regular re-evaluation
- Procedures	Traumatic Repetitious	Early central venous line access Use distraction methods and child-adapted information
- Psychosocial	Anxiety Parents in despair	Family-centred care approach; social worker should be invited in the care of the family
Skin/tissue- Access	Burning, rashes, collapsed veins	Cold blankets Central venous line
- Sleep	Disturbed due to management of symptoms and treatment	Coordinate procedures and drug administrations to minimise the number of times the child’s room is entered during the night

### Strengths and limitations

Using both quantitative and qualitative methodology may create valuable insights, and the different approaches enable the creation of new knowledge [32]. When conducting such design, it can be challenging to combine the paradigms of qualitative and quantitative nursing research [33]. However, we would argue that this is positive in our study since the results from the quantitative audits of clinical nursing records and qualitative interview data complements each other. Focus group interviews are valuable when it comes to describing people’s experiences and attitudes [34]. The objective of focus group interviews is to receive high-quality data in a social context where the participants can reflect on their own views in relation to others [14]; we believed this goal was met. To ensure trustworthiness data analysis was performed using researcher triangulation and quotes are used to illustrate the findings.

A possible limitation is the data obtained via clinical nursing records. It may be, for example, that different RNs interpreted a child’s symptoms differently and therefore documented it differently.

## Conclusion

The results show that children suffering from MIS-C have many different areas of nursing focus, which challenges RNs and other professionals in paediatric hospital care. RNs, together with the interprofessional team, should pay attention to the quick changes in children’s health status. The care of children suffering from MIS-C demands resources and time from HCPs, especially RNs, to be able to meet the children’s needs and reduce their illbeing. Dissemination of the recommendations based on nursing status and observations in this study may contribute to improved quality of nursing care.

## Supplementary Information

**Additional file 1.**

## Data Availability

The datasets used and/or analysed during the current study are available from the corresponding author upon reasonable request.

## Abbreviations

HCP: Health Care Professionals
MIS-C: Multisystem inflammatory syndrome in children
RN: Registered Nurses
PVC: Peripher vein catheter
