# Supplementary material for: Multi-inflammatory syndrome in children (MIS-C) associated with COVID-19: a nursing perspective experience report from a high-income tertiary paediatric hospital context
Source: Pediatr Rheumatol Online J. 2023 Jan 18;21:5. doi: 10.1186/s12969-023-00786-y (PMC9845810; doi:10.1186/s12969-023-00786-y)
Supplement: Supplementary file 1 — Additional file 1. [file 12969_2023_786_MOESM1_ESM.docx]

**Question guide used in focus group interviews with nurses who cared for children with Multisystem inflammatory syndrome (MIS-C)**

----------------------------------------------------------------------------------------------------

Question guide:

Introduction:

Thank you for coming.

Background to the project…

Open questions:

- What is your experience of caring for children with (suspected) MIS-C

- How do you feel that parents and other relatives experience having a child with MIS-C?

- What have you learned from caring for these children?

Probing questions:

- Can you tell us more? What do you think about that?

- What do you mean, can you please explain further? Can you please give examples?

? What re-measures / plans?

Additional questions:

- Which care-specific areas do you think have been important?

- Which care-specific measures do you think have been important / difficult / challenging?

- Have you discovered any red flags while caring for the children?

**Question guide related to the VIPS-model:**

Could you please tell us how you experience the following specific nursing concepts when caring for children suffering from MIS-C and please also tell us what nursing activities you have conducted related to each concept.

**Table 1: Question areas**

| VIPS status | Symptoms | Activities |
| --- | --- | --- |
| Breathing |  |  |
| Circulation: |  |  |
| Nutrition: |  |  |
| Pain: |  |  |
| Elimination: |  |  |
| Skin: |  |  |
| Psychosocial: |  |  |
| Coordination: |  |  |
| Drug administration |  |  |
| Sampling |  |  |
|  | | |
